# Older children are at increased risk of *Plasmodium vivax* in south-central Ethiopia: a cohort study

**DOI:** 10.1186/s12936-021-03790-3

**Published:** 2021-06-06

**Authors:** Taye Gari, Tarekegn Solomon, Bernt Lindtjørn

**Affiliations:** 1 School of Public Health, College of Medicine and Health Sciences, Hawassa University, Hawassa, Ethiopia; 2 Centre for International Health, University of Bergen, Bergen, Norway

**Keywords:** *Plasmodium vivax*, Mosquito breeding site, Risk, Cohort, Ethiopia

## Abstract

**Background:**

Better understanding of the distribution of *Plasmodium vivax* and its risk factors could be used to prevent and control malaria infection. Therefore, the aim of this study was to characterize the distribution and risk factors of *P. vivax,* and to compare them with *Plasmodium falciparum* occurrence in south-central Ethiopia.

**Methods:**

A cohort of 34,548 individuals were followed for 121 weeks between 2014 and 2016 as part of larger cluster randomized controlled trial to evaluate the effect of long-lasting insecticidal nets (LLINs) and indoor residual spraying (IRS) on malaria prevention in Ethiopia. Weekly home visit (active search) and patient self- report to health post (passive search) between the weekly home visits were used to register malaria cases. A blood sample was collected by finger prick and malaria was diagnosed using rapid diagnostic test (RDT). Generalized estimating equation (GEE) Poisson model that accounts for repeated measure of malaria episodes was applied to assess the risk factors of *P. vivax* episode.

**Results:**

The overall incidence rate of *P. vivax* was 7.4 episodes per 1000 person-years of observation. The study showed households closer to the lake Zeway and Bulbula river (potential mosquito breeding sites) were more at risk of *P. vivax* infection (incidence rate ratio (IRR): 1.33; 95% CI = 1.23–1.45)*.* Furthermore, the age group under 5 years (IRR: 1.40, 95% CI = 1.10–1.79), the age group 5–14 years (IRR: 1.27, 95% CI = 1.03–1.57), households with less educated household head (IRR: 1.63, 95% CI = 1.10–2.44) and house roof made of thatch/leaf (IRR: 1.35, 95% CI = 1.11–1.65) were at higher risk for *P. vivax.* Similar explanatory variables such as distance from the breeding sites, age group (under 5 years but not 5–14 years old), educational status and type of housing were also found to be the predictors of *P. falciparum* incidence.

**Conclusion:**

Households living closer to a mosquito breeding site, age group under 15 years, less educated household heads and thatch/leaf roof housing were the risk factor for *P. vivax.* The result of this study can be used for tailored interventions for malaria control and prevention by prioritizing those living close to potential mosquito breeding site, enhancing bed net use of children less than 15 years of age, and improving housing.

**Supplementary Information:**

The online version contains supplementary material available at 10.1186/s12936-021-03790-3.

## Background

Malaria is an infectious disease mainly transmitted by female *Anopheles* mosquito bite [1]. Globally, an estimated 229 million malaria cases and 409,000 deaths were reported in 2019. The majority of the cases (94%) and deaths (94%) were reported from Africa [2]. The same report showed that *Plasmodium vivax* accounts for about 3% (5 million) of all the estimated cases. More than 85% of the global *P. vivax* cases occurred in six countries: India, Afghanistan, Pakistan, Ethiopia, Papua New Guinea and Indonesia [2, 3].

Malaria is caused by five species of *Plasmodium *(*P. vivax, Plasmodium falciparum, Plasmodium malariae, Plasmodium ovale* and *Plasmodium knowlesi*) [4]. *Plasmodium falciparum* is a cause of severe malaria, and most prevalent (99.7% of malaria cases in 2018) in Africa [5]. Whereas, *P. vivax* has the widest geographic distribution of the four malaria parasites, and can also cause severe malaria in children [3, 6]. Although, the proportion of *P. vivax* in most of sub-Saharan Africa is low (1%), it accounts for about 40% of malaria cases in Ethiopia and outside Africa, mainly Latin America and Asia [7].

In Ethiopia, *P. vivax* (40%) and *P. falciparum* (60%) are the two dominant causes of malaria infection [8]. The trend in malaria cases and deaths has substantially declined between 2000 and 2015 following the rapid massive scale up of malaria diagnosis and treatment and application of vector control tools, such as long-lasting insecticidal nets (LLINs) and indoor residual spraying (IRS) [9, 10]. However, evidences show that the rate of malaria case has either stalled or even increased in some areas within the country between 2015 and 2018 [5]. For example; in Southern Nations and Nationalities Regional State of Ethiopia an increase in confirmed malaria cases for the months between July and August 2019 was reported compared to same months in 2018 [11].

Studies have shown that altitude [12, 13], age [14], wealth status [15, 16], population movement and proximity to mosquito breeding place [14] are the risk factors for *P. vivax* infection. However, the observed relationship particularly between distance of living house from mosquito breeding site and *P. vivax* transmission varied from one area to another, and need further investigation. For example, some reported increased risk of *P. vivax* in individuals living closest to breeding site [14], other reported increased risk of *P. vivax* with increase in distance from breeding site [12], and other study has shown absence of association between breeding site and *P. vivax* [17]*.*

In areas where the two infections co-exist, the incidence of *P. vivax* declines slower than that of *P. falciparum* infection, and remains the main cause of malaria [10]. *Plasmodium vivax* is more difficult to eliminate than *P. falciparum.* This could be because *P. vivax* remains dormant in human liver for weeks, months or years, the source of relapse infection; the parasite develops in the vector at lower temperatures than *P. falciparum,* and it has wider geographic distribution and extending to highland areas [18]. Moreover, unlike *P. falciparum,* the gametocytes of *P. vivax* are released into the bloodstream as soon as a patient becomes ill. Therefore, a patient infected with *P. vivax* transmits the parasite to mosquitoes before the clinical symptoms appear [18]. In a previous study, Solomon et al. evaluated spatio-temporal clustering and risk factors of clustering for *P. falciparum* and *P. vivax* using data from cluster randomized controlled malaria prevention trial (the same data source as the current study) [19]. The understanding of the distribution of *P. vivax* and risk factors could be used to prevent and control *P. vivax* malaria infection. Therefore, the objective of this study was to characterize the distribution and risk factors for *P. vivax* compared to *P. falciparum* in the Great Rift Valley of Ethiopia, from a large cluster randomized controlled malaria prevention trial carried out in south-central Ethiopia.

## Methods

### Study setting, design and participants

**Fig. 1:**
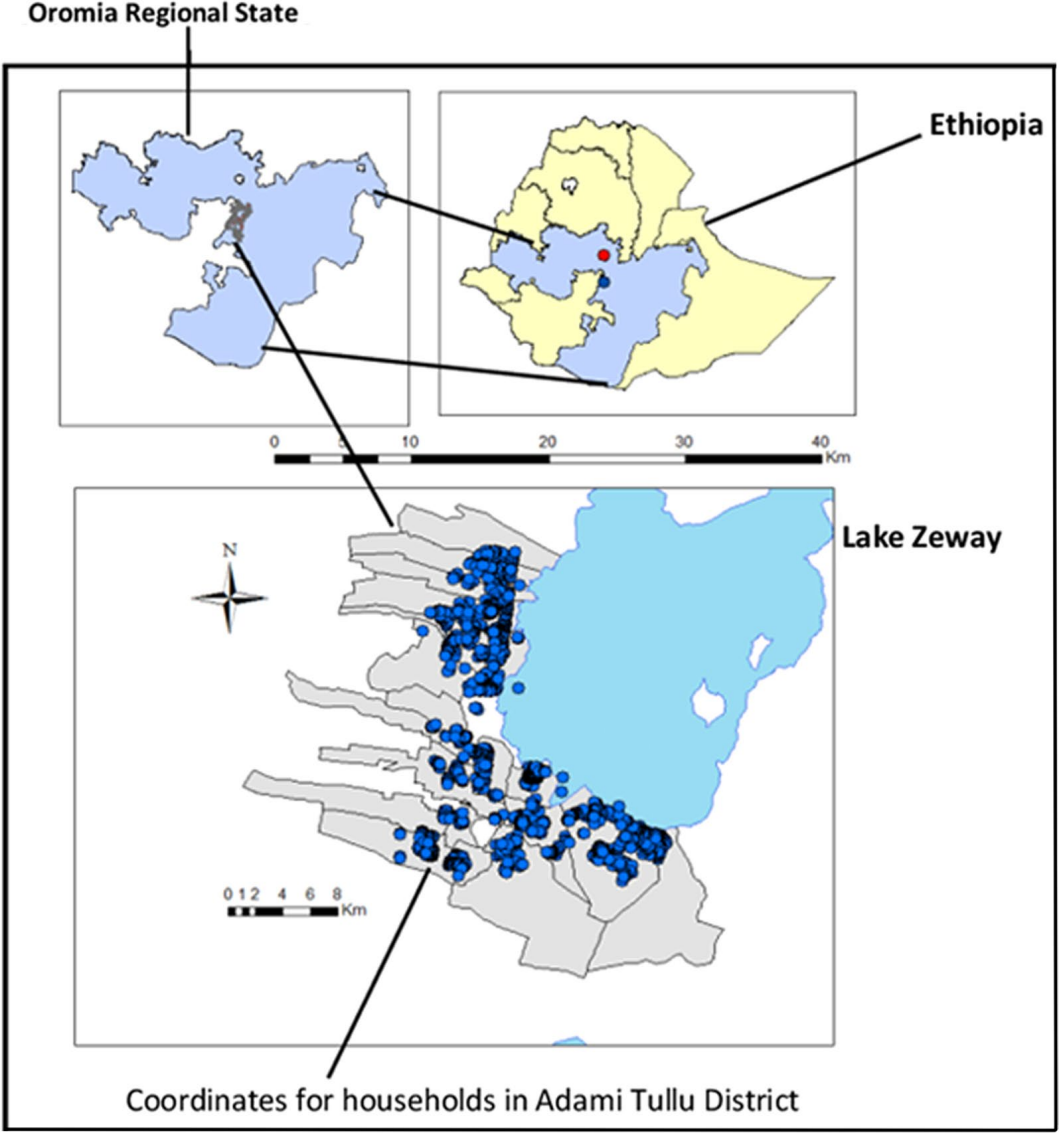
Map of the study area with location of households in Adami Tullu District in south-central Ethiopia

The study was conducted in rural area of Adami Tullu district in the Oromia Regional State in Ethiopia. The capital of the district, Zeway (or Batu) is located about 160 km south of the capital Addis Ababa at latitude of 7°56′N and longitude of 38°42′E with an altitude of 1600 m above sea level. According to the 2007 National Census [20], the projected total population of the district for the year 2014 was 173,000. The district has 48 *kebeles* (the lowest government administrative unit), and a *kebele* is further divided into villages (“Gare”). Lake Zeway, one of the Ethiopian Rift Valley lakes borders the district. The residents use Lake Zeway and Bulbula River (coming out of Lake Zeway) for irrigation to cultivate crops. Furthermore, there is a large flower farm that use water from the lake and the river. Such water based development were reported to be potential malaria vector breeding sites [21]. Malaria is among a common public health problem in the district. There is one health post deployed with two female health extension workers in each *kebele*. The health extension workers provide malaria diagnosis and treatment, prevention and control services including basic health services (Fig. 1).

This cohort study is a part of large cluster randomized controlled trial to evaluate the effect of LLINs and IRS on malaria prevention in Ethiopia, short named “MalTrials project”. More information about the details of the design, participants and some results have been published elsewhere [16, 19, 22–25]. In summary, villages within 5 km of the Lake Zeway and Bulbula River in 13 *kebeles* were randomly selected. Distance of the households from the lake and river border (potential mosquito breeding place) was calculated using a proximity analysis tool in ESRI ArcMap 10.3.1. A village or cluster has on average population size of 175 residents. The villages or *Gare* (local name for village) were randomly assigned to the four malaria intervention arms (IRS + LLINs, LLINs alone, IRS alone and Routine).

### Sample size estimation

As presented elsewhere [22], the sample size calculation method for cluster randomized controlled trial that accounts for intra-cluster correlation coefficient, malaria incidence rate, estimated effect size of the intervention, power and confidence level was used to calculate the sample size. The assumptions used for sample size calculation were: long-lasting insecticidal nets (LLINs) utilization and indoor residual spraying (IRS) independently reduce malaria incidence by around 50%, and the combined use of LLINs and IRS could reduce malaria incidence by 75%. The intra-cluster correlation coefficient was obtained from a pilot study conducted from September to December 2013 in villages around the Lake Zeway in Ethiopia. A total of 34,548 individuals in 176 villages randomized into four arms with 44 villages, each (LLINs + IRS, IRS alone, LLINs alone, and control or routine practice) were followed for 121 weeks. The control arm received the standard routine practice of malaria prevention and control of the Ethiopian Malaria Prevention and Control Programme [26].

### Data collection

Census and weekly data were collected by diploma graduates non health professionals. Blood sample collection for malaria diagnosis and treatment was carried out by diplomaed nurses. The data collectors and nurses were trained before starting the trial and also refresher training was given periodically during the follow-up study. The structured questionnaires were obtained from malaria study conducted at Chano Mille in south Ethiopia [15]; were modified, pre-tested and used in this study (translated into local language, haemoglobin and malnutrition survey questions included).

### Baseline and census

All households within 5 km of Lake Zeway from 13 *kebles* were enumerated. Identification number was given to the houses using metal plates, and unique number was given for all household members. Baseline data were collected to list household members, socio-demographic, economic, history of malaria illness and malaria prevention. Furthermore, the household geographic coordinate was taken using a Global Positioning System (GPS) device (Garmin GPSMAP60CSx, Garmin International Inc., Olathe, KS, USA). The census was repeated after one year to enumerate those people newly joined the cohort (birth, marriage and migration) and left the cohort (death and lost to follow-up).

### Weekly data

Weekly home visits (active case search) were carried out to identify person with clinical sign of malaria and to assess LLINs utilization practice by the household members. Any person identified with the history of fever within the previous 48 h during the home visit was referred to health post with referral slip. Moreover, family members were advised to visit health post if encounter fever (passive case search) between the weekly visit.

### Malaria diagnosis

Blood sample from patients presented with history of fever within the previous 48 h was taken using finger prick by trained clinical nurses employed by the MalTrials project. Rapid diagnostic test (RDT) was performed in the health post using CareStart^®^ Malaria Pf/Pv combo test (Access Bio, Inc., Somerset, NJ, USA).

### Statistical analysis

Data were entered and cleaned using SPSS version 24 (SPSS Inc, Chicago, USA), and analysed using STATA version 15 (StataCorp, Texas, USA). Descriptive statistics was carried out to summarize the data. A principal component analysis method [27] was used to construct household wealth index from 14 variables related to household assets and livestock ownership [28]. The constructed index was used to group the households into rich, middle and poor socioeconomic classes. The incidence rate of *P. vivax* or *P. falciparum* was calculated by dividing number of each species episodes per 1000 person years of observation. ArcMap 10.3.1 software was used to calculate the distance in kilometres between household and nearest main potential mosquito breeding site (the shore of Lake Zeway and Bulbula river) [19]. The outcome variable, *P. vivax* or *P. falciparum* episode was count variable that follows a Poisson distribution, and to account for the within subject correlation due to repeated measurements of malaria status generalized estimating equation (GEE) was applied. The GEE procedure is the extension of general linear model [29]. The repeated assessment of malaria status within one subjects are not independent of each other and, hence, GEE accounts for within-subject correlations. In the GEE model, Poisson log-linear link function was the specified probability, and exchangeable was the working correlation matrix structure. Pearson chi-square (χ^2^) was the scale parameter, and robust estimator was the specified covariance matrix. The term used to build the reported model was the main effect, and the parameter estimation method was a hybrid with a maximum Fisher scoring iteration of 1. Finally, Kernel was specified for the log quasi-likelihood function. The potential predictors of *P. vivax* or *P. falciparum* were age group, gender, educational status of the head of households, wealth status, intervention arms, distance from mosquito breeding place, and roof of the housings. The association between *P. vivax* or *P. falciparum* and the predictor variables was assessed using bivariable and multivariable Poisson log-linear model, and incidence rate ratio (IRR) with 95% confidence interval was reported.

## Results

In this study 34,548 individuals from 6071 households were followed. Half (50.2%) of the participants were male, and in the age group of 15 years and above (50%). Fifty one percent of the participants were illiterate, ~ 33.2% were poor, 69% of them had more than 5 household size, around 26% of the households received both LLINs and IRS, and median (interquartile range) of household distance from breeding site was 1.66 (0.70–2.95) km (Table 1).

**Table 1:** Socio-demographic characteristics of study subject south-central Ethiopia, October 2014 to January 2017

Variables (n = 34,548)	Number	Percent
Sex		
Male	17,327	50.2
Female	17,221	49.8
Age group in years		
< 5	6488	18.8
5–14	11,136	32.2
≥ 15	16,924	49.0
Educational status of household head		
Illiterate	19,752	51.2
Read and write	3,738	10.8
Primary education complete	8,155	23.6
Secondary and above	2,903	8.4
Wealth Status		
Poor	11,459	33.2
Middle	11,578	33.5
Rich	11,511	33.3
Family size		
≤ 5	10,664	30.9
> 5	23,884	69.1
Intervention arms		
IRR + LLIN	9104	26.4
LLIN alone	8038	23.3
IRS alone	8567	24.8
Routine	8839	25.5
Median (IQR) distance from mosquito breeding site	1.66 (0.70–2.95) km	

*IQR* interquartile range, *IRS* indoor residual spraying, *LLINs* long lasting insecticidal nets

### Incidence of *P. vivax* and *P. falciparum*

**Fig. 2:**
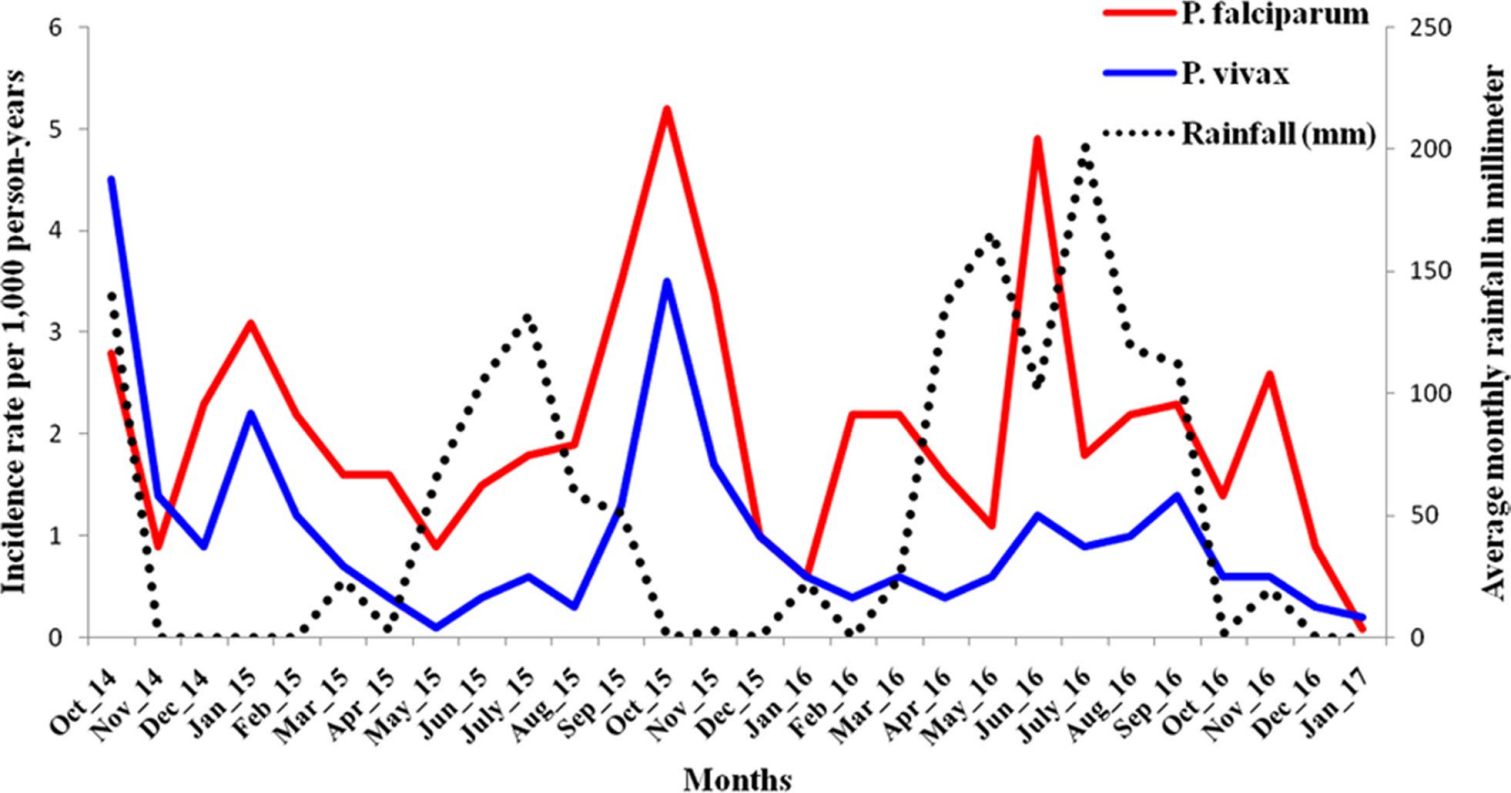
Malaria infection rates, and monthly rainfall during the study period per 1000 person-years south-central Ethiopia, October 2014 to January 2017

Overall, 531 episodes of *P. vivax* including mixed infection in 487 participants were observed during the follow-up period. Out of the 487 participants, 450 developed one episode, 29 developed 2 episodes, six experienced 3 episodes and one experienced 5 episodes of *P. vivax*. On the other hand, 884 *P. falciparum* including mixed infection episodes occurred during the whole study period. The total number of *P. falciparum* episode experienced by the individuals was one episode (753 individuals), two episodes (56 individuals), three episodes (6 individuals) and four episodes (1 individual). As shown in Fig. 2, malaria occurs in the study area throughout the year with varying intensity dominated by *P. falciparum*. The incidence rate of *P. falciparum* and *P. vivax* was higher during the major malaria transmission seasons (September–December).

The overall incidence rate of *P. vivax* was 7.4 episodes per 1000 person-years of observation. *Plasmodium vivax* incidence was high among children under 5 years old (9.6 per 1000 person-years), less educated (read and write) household heads (11.4 per 1000 person-years), house roof made of thatch or leaf (7.9 per 1000 person-years) and living within 1 km from mosquito breeding site (10.8 per 1000 person-years). Whereas, the overall incidence rate of *P. falciparum* was 12.3 episodes per 1000 person-years of observation. The incidence of *P. falciparum* was high among children under 5 years old (15.9 per 1000 person-years), less educated (read and write) household heads (10.88 per 1000 person-years), living within 1 km from mosquito breeding site (17.00 per 1000 person-years) and house roof made of thatch or leaf (14.22 per 1000 person-years) (Table 2).

**Table 2:** Incidence rate and incidence rate ratio (IRR) of *P. vivax* and *P. falciparum*, south-central Ethiopia, October2014 to January 2017

Variables (n = 34,548)		Person-years	*P. vivax*			*P. falciparum*		
			No. of episode	IR/1000 PY	IRR	No. of episode	IR/1000 PY	IRR
All		71,859.1	531	7.39		884	12.30	
Sex								
Male		36,180.4	259	7.16	1.00	445	12.30	1.00
Female		35,678.7	272	7.62	1.06	439	12.30	1.00
Age group in years								
< 5		12,739.8	122	9.58	1.37	203	15.93	1.12
5–14		23,727.3	184	7.75	1.11	275	11.59	0.81
15–24		13,999.7	76	5.43	0.78	101	7.21	0.51
≥ 25		21,392.3	149	6.97	1.00	305	14.26	1.00
Educational status of household head								
Illiterate		41,303.9	288	6.97	1.03	457	11.06	0.99
Read and write		7712.3	88	11.41	1.69	128	16.60	1.49
Primary education		16,918.3	115	6.80	1.01	233	13.77	1.24
Secondary and above		5924.7	40	6.75	1.00	66	11.14	1.00
Wealth Status								
Poor		23,837.3	171	7.17	1.05	301	12.63	1.10
Middle		23,994.1	196	8.17	1.20	308	12.84	1.12
Rich		24,027.7	164	6.83	1.00	275	11.45	1.00
Family size								
≤ 5		22,036.5	164	7.44	1.01	280	12.71	1.05
> 5		49,822.6	367	7.37	1.00	604	12.12	1.00
Intervention arms								
IRR + LLIN		18,712.6	143	7.64	0.97	236	12.61	1.00
LLIN alone		17,243.0	105	6.09	0.78	209	12.12	0.96
IRS alone		17,152.1	136	7.93	1.01	222	12.94	1.03
Routine		18,751.4	147	7.84	1.00	217	11.57	0.92
House roofing								
Thatch/grass		33,481.5	266	7.94	1.15	476	14.22	1.12
Corrugated iron		38,377.6	265	6.91	1.00	486	12.66	1.00
Distance from breeding								
≤ 1.66 km		35,330.7	337	9.54	1.80	486	16.97	1.48
> 1.66 km		36,528.4	194	5.31	1.00	398	11.50	1.00

^‡^*PY* person years, ± *IR* incidence rate, #*IRR* incidence rate ratio, €*IRS* indoor residual spraying, ¥*LLINs* long lasting insecticidal nets, $ *km* kilometre

### Risk factors of *P. vivax* and *P. falciparum* episodes

Multivariable generalized Poisson log–linear model analysis was fitted to measure the risk factors for *P. vivax* and *P. falciparum.* The result has shown that the same variables, distance of household from mosquito breeding site, age groups, educational status of household head and roof of housing were the risk factors for both *P. vivax* and *P. falciparum*. In this study, after controlling for potential confounding factors households closer to mosquito breeding place were more at risk of *P. vivax* infection (IRR: 1.33; 95% CI = 1.23–1.45)*.* Furthermore, age group under 5 years (IRR: 1.40, 95% CI = 1.10–1.79), age group 5–14 years (IRR: 1.27, 95% CI = 1.03–1.57) and less educated (read and write) household head (IRR: 1.63, 95% CI = 1.10–2.44) were the predictors of *P. vivax.* The study also showed that peoples living in a house roof made of thatch/leaf were more at risk of experiencing *P. vivax* (IRR: 1.35, 95% CI = 1.11–1.65) than corrugated iron roof housing (Additional file 1: Table S3). Unlike *P. vivax*, age group 5–14 years was not a risk factor for *P. falciparum.*

## Discussion

This cohort study followed 34,548 participants in a drought prone rural south-central Ethiopia for 121 weeks. Overall, 531 episodes of *P. vivax* were observed. The incidence rate per 1000 persons-years of observation was about 7.4 episodes for *P. vivax* and 12.3 episodes for *P. falciparum.* The study showed seasonal variation in malaria transmission for both *P. vivax* and *P. falciparum.* The observed risk factors for *P. vivax* and *P. falciparum* were distance of household from mosquito breeding site, age group, educational status of household head and roof of housing.

The observed incidence rate of *P. vivax* in this study (7.4 episodes per 1000 persons-years of observation) was lower than a pilot study that was conducted prior to the main trial in the same study area from August 2013 to December 2013, in which the average incidence rate was 4.6 episodes per 10,000 person-weeks of observation (approximately 24 episodes per 1000 person-years) [30], and study conducted in Arba Minch, south Ethiopia (IR = 12.3/1000 person-years of observation) [14]. This could be due to difference in duration of follow up, the pilot study was conducted during the 16 weeks of major malaria transmission season, and the low malaria incidence in this study could be related to severe shortage of rain and drought that affected the study area [28].

In this study household proximity to potential mosquito breeding site was the risk factor for both *P. vivax* and *P. falciparum*. The presence of water bodies, where female mosquitoes lays their eggs, is critical for the life of the mosquito [31]. The female *Anopheles* mosquito carrying eggs need human or animal blood and thus visit nearby houses to take their blood meal. Previous studies reported higher mosquito density in households closest to mosquito vector breeding areas [30, 32]. Therefore, individuals living close to mosquito breeding site are at higher risk of infection than those living far-away from breeding site. Similar cohort study from rural southern Ethiopia by Nissen et al. showed that households close to breeding site are 3.5 times more at risk to *P. vivax* infection than the group furthest away [14]. Furthermore, a cohort study by Loha et al. from similar setting in Chano Mille rural southern Ethiopia also showed that household proximity to breeding place is a risk factor of *P. falciparum* [15]. Another survey conducted at Gilgel-Gibe hydro-electric dam in Ethiopia also showed higher odds of *P. vivax* among children living close to the dam [33]. Similar study from Uganda reported increased risk of *Plasmodium* infection among residents living close to rice growing areas [34]. In contrary to the current study, some local studies reported increased risk of *P. vivax* infection among residents living far from dam [35]. This could be explained by the difference in measuring distance from mosquito breeding area, unlike the previous studies, which used the distance from the dam in this study the main water bodies in the study areas were identified and used as proxy for vector breeding site.

On the other hand, children under the age of 5 years and 5–14 years old were more at risk of *P. vivax* compared to those in the age group of 15 years and above. In study from the same setting and time period by Solomon et al. demonstrated that those in the age group 15 year and above use LLINs more frequently compared to those less than 5 years old [24]. This LLIN usage practice and immunity could have decreased the risk of malaria infection in the older age groups.

When malaria incidence declines, and both species co-exist, *P. vivax* incidence decreases more slowly than *P. falciparum* [7]. Also, in a situation where malaria incidence has declined from high to low incidence, the overall proportion of *P. vivax* increases and becomes the dominant causes of malaria. In line with earlier finding [36], this study showed increased risk of *P. vivax* but not *P. falciparum* among children 5–14 years of age.

In this study, materials from which the roof of the house made, thatch/leaf was observed as risk factor for *P. vivax* infection. This could be due to the presence of eves, poorly fitted doors, and holes in walls of roofs made of thatch/leaf. Studies have shown that modern houses, roof made of corrugated iron and others are associated with decreased risk of malaria infection by reducing mosquito entry to house [37].

Furthermore, less educated household heads (read and write) were more at risk for *P. vivax* infection than those who had received a formal education. This could be explained by the fact that household heads with formal education better understand and use malaria prevention tools, that could reduce risk of infection than less educated household heads. Study from same setting in the same time period has shown that household heads with a formal education were more likely to use LLINs compared to less educated ones [24].

This cohort study was based on a random selection of clusters or villages from rural community for cluster randomized controlled trial. The study involved large sample with adequate power for long follow up (121 weeks) period in a drought prone area. Even though the sample is representative of a population in a similar setting in Ethiopia’s rift valley, the generalizability of this study may be harmed by the extreme drought that hit the study region. Therefore, this circumstance should be taken into account while interpreting the finding of this study. The other limitation is that it was not established whether a *P. vivax* episode was a new infection or a relapse. In the event of relapse, the *P. vivax* infection detected may be months or years old. Therefore, lack of knowledge on the actual place of residence at the time of initial infection could lead to overestimation of malaria episode in evaluating the association between distances from mosquito breeding site and *P. vivax* episodes.

## Conclusion

In this study, household living closer to a mosquito breeding site, age group under 15 years, less educated household heads and thatch/leaf roof housing were the risk factor for *P. vivax.* The result of this study can be used for tailored interventions for malaria control and prevention by prioritizing those living close to potential mosquito breeding site, enhancing bed net use of children less than 15 years of age, and improving housing.

## Supplementary Information

**Additional file 1**: **Table S3** Risk factors of *P. vivax *and *P. falciparum *episodes Generalized log-linear model, Adami Tullu District, south-central Ethiopia.

## Data Availability

The datasets used in this study can be available from the corresponding author upon the reasonable request. **Ethics approval and consent to participate** Ethical clearance for the trial was obtained from Institutional Review Board of College of Health Sciences at Addis Ababa University, the Ethiopian Ministry of Science and Technology (ref: 3.10/446/06) and the Regional Committee for Medical and Health Research Ethics, Western Norway (ref: 2013/986/REK Vest). The trial was registered at the Pan African Clinical Trials Registry under the registration number PACTR201411000882128 online on 8 September 2014. Informed verbal consent was obtained from head of households during baseline and follow up census, and from all individuals.

## Abbreviations

GEE: Generalized estimating equation
IQR: Interquartile range
IRR: Incidence rate ratio
IRS: Indoor residual spraying
LLINs: Long-lasting insecticidal nets
RDT: Rapid diagnostic test
