# Supplementary material for: Older children are at increased risk of Plasmodium vivax in south-central Ethiopia: a cohort study
Source: Malar J. 2021 Jun 6;20:251. doi: 10.1186/s12936-021-03790-3 (PMC8183059; doi:10.1186/s12936-021-03790-3)
Supplement: Supplementary file 1 — Additional file 1: Table S3 Risk factors of P. vivax and P. falciparum episodes Generalized log-linear model, Adami Tullu District, south-central Ethiopia. [file 12936_2021_3790_MOESM1_ESM.docx]

**Table 3 Risk factors of *P. vivax* and *P. falciparum* episodes Generalized log-linear model, Adami Tullu District, south-central Ethiopia**

| **Variables** | ***Plasmodium vivax*** | | | ***Plasmodium falciparum*** | | |
| --- | --- | --- | --- | --- | --- | --- |
|  | **Bivariable** | ***Multivariable*** |  | **Bivariable** | ***Multivariable*** |  |
|  | **IRR**€ **(95% CI)** | **IRR**€ **(95% CI**‡**)** | ***P-value*** | **IRR**€ **(95% CI**‡**)** | **IRR**€ **(95% CI)** | ***P-value*** |
| **Sex** |  |  |  |  |  |  |
| Female | 1 | 1 |  | 1 | 1 |  |
| Male | 0.93 (0.77 - 1.12) | 0.93 (0.77 - 1.12) | 0.433 | 1.01 (0.88 - 1.17) | 1.01 (0.88 - 1.16) | 0.90 |
| **Age group in years** | |  |  |  |  |  |
| < 5 | 1.42 (1.12 - 1.81) | 1.40 (1.10 - 1.79) | 0.006 | 1.31 (1.10 - 1.57) | 1.27 (1.06 - 1.51) | 0.008 |
| 5 – 14 | 1.26 (1.02 - 1.55) | 1.27 (1.03 - 1.57) | 0.026 | 1.03 (0.87 - 1.22) | 1.03 (0.88 - 1.22) | 0.69 |
| ≥ 15 | 1 | 1 |  | 1 | 1 |  |
| **Education of household head** | |  |  |  |  |  |
| Illiterate | 1.06 (0.75 - 1.50) | 1.09 (0.77 - 1.56) | 0.622 | 1.02 (0.77 - 1.34) | 1.02 (0.77 - 1.34) | 0.912 |
| Read and write | 1.71 (1.15 - 1.54) | 1.63 (1.10 - 2.44) | 0.016 | 1.52 (1.10 - 2.09) | 1.46 (1.06 - 2.01) | 0.022 |
| Primary | 1.01 (0.70 - 1.48) | 1.00 (0.69 -1.46) | 0.989 | 1.26 ( 0.94 - 1.69) | 1.24 (0.93 - 1.67) | 0.145 |
| Secondary and above | 1 | 1 |  | 1 | 1 |  |
| **Household size** |  |  |  |  |  |  |
| ≤ 5 person | 0.98 (0.79 - 1.22) | 0.97 (0.78 - 1.20) | 0.780 | 1.03 (0.89 - 1.21) | 1.02 (0.88 - 1.19) | 0.775 |
| > 5 person | 1 | 1 |  | 1 | 1 |  |
| **Wealth Index** |  |  |  |  |  |  |
| Poor | 1.09 (0.87 - 1.37) | 0.91 (0.72 - 1.14) | 0.42 | 1.10 (0.93 -1.31) | 1.00 (0.84 - 1.20) | 0.968 |
| Middle | 1.20 (0.96 - 1.51) | 1.09 (0.87 - 1.37) | 0.447 | 1.11 (0.93 - 1.32) | 1.06 (0.89 - 1.26) | 0.539 |
| Rich | 1 | 1 |  | 1 | 1 |  |
| **Roof of House** |  |  |  |  |  |  |
| Thatch/Leaf | 1.16 (0.97 - 1.41) | 1.35 (1.11 - 1.65) | 0.003 | 1.18 (1.03 - 1.36) | 1.30 (1.13 - 1.51) | <0.001 |
| Corrugated Iron | 1 | 1 |  | 1 | 1 |  |
| **Intervention arms** | |  |  |  |  |  |
| LLINS + IRS | 1 | 1 |  | 1 | 1 |  |
| LLINs ¥ | 0.80 (0.61 - 1.05) | 0.80 (0.61 - 1.06) | 0.118 | 1.00 (0.83 - 1.22) | 1.00 (0.82 - 1.22) | 0.997 |
| IRS€ | 0.99 (0.78 - 1.28) | 1.05 (0.82 - 1.34) | 0.715 | 0.99 (0.82 - 1.22) | 1.04 (0.85 - 1.26) | 0.728 |
| Routine | 1.02 (0.80 - 1.31) | 1.10 (0.86 - 1.41) | 0.437 | 0.95 (0.78 - 1.16) | 0.99 (0.82 - 1.21) | 0.956 |
| **House distance from breeding site** | 1.30 (1.20 - 1.40) | 1.33 (1.23 - 1.45) | <0.001 | 1.18 (1.12 - 1.24) | 1.19 (1.13 - 1.26) | <0.001 |

‡CI= Confidence Interval; ±IRR= Incidence Rate Ratio; €IRS= Indoor Residual Spraying; ¥LLINs= Long Lasing Insecticidal Nets
